# Sperm subpopulations differing in mitochondrial abundance show divergent nuclear allele frequencies

**DOI:** 10.1371/journal.pone.0358219

**Published:** 2026-09-15

**Authors:** Tyler Weide, James Koltes, Juan Steibel, Karl Kerns

**Affiliations:** 1 Department of Animal Science, Iowa State University, Ames, Iowa, United States of America; 2 Interdepartmental Genetics and Genomics, Iowa State University, Ames, Iowa, United States of America; University of Hyderabad, INDIA

## Abstract

Mammalian ejaculates contain heterogeneous sperm subpopulations that differ in subcellular architecture and developmental history, despite appearing morphologically uniform. The extent to which this cellular heterogeneity reflects underlying nuclear genomic structure within a sire remains largely unexplored. Mitochondrial architecture in sperm is established during spermatogenesis, with final assembly and organization occurring during spermiogenesis under nuclear genomic control, positioning variation in mitochondrial abundance and organization as a potential phenomic indicator of within-sire allelic segregation. Here, we tested whether sperm subpopulations defined by differing mitochondrial abundance exhibit systematic differences in nuclear allele representation. Boar sperm were resolved into low and high mitochondrial subpopulations using fluorescence-activated cell sorting based on MitoTracker™ Green fluorescence while excluding debris, doublets, and non-viable cells. Epifluorescence microscopy confirmed that high MitoTracker™ Green fluorescence sperm possessed longer mitochondrial sheaths, validating a structural distinction between subpopulations. Whole-genome sequencing of paired mitochondrial subpopulations from three boars was performed, and allelic ratio distortion was evaluated relative to heterozygous baseline populations. Analyses across heterozygous loci genome-wide identified candidate allele frequency shifts between mitochondrial-defined subpopulations, suggesting non-random segregation of alleles within ejaculates. Using a minimum sequencing depth of 30 reads in both sorted fractions, 182 candidate SNPs were identified with evidence of allele-frequency differences between mitochondrial fluorescence-defined subpopulations. These findings suggest that sperm mitochondrial abundance can potentially serve as an indirect, high-throughput marker of nuclear genomic heterogeneity within sires. This proof-of-concept framework establishes a foundation for future studies integrating sperm phenotyping, genome-wide allele-frequency analysis and functional validation to better characterize gamete-level heterogeneity.

## Introduction

Mammalian ejaculates comprise highly heterogeneous sperm populations that differ in subcellular architecture [1], metabolic organization [2,3], and developmental history [4,5], although appearing morphologically uniform at the light microscopic level. Despite this apparent uniformity, ejaculated sperm are highly diverse in metabolic status [6]. This heterogeneity reflects differences that originate during spermiogenesis [7], epididymal maturation [5,8,9], and post-ejaculatory modification [10–12], resulting in discrete sperm subpopulations that represent alternative cellular realizations of a sire’s genome rather than uniform carriers of genetic material. Rather than serving solely as determinants of fertilization success, these subpopulation-level differences provide a phenomic window into how nuclear genetic variation is structured, expressed, and partitioned among gametes within a single individual. One major contributor to this functional heterogeneity is the mitochondrion [13], which represents a complex, nuclear-controlled organelle [14,15] whose abundance [16], organization [17], and spatial arrangement [18] are established during spermatogenesis and spermiogenesis. Mitochondria play a central role across mammalian sperm physiology by supplying ATP for motility [19], regulation of reactive oxygen species (ROS) [20], and supporting capacitation-associated signaling events [21,22]. Importantly, the mitochondrial sheath of sperm is assembled under direct nuclear genomic control [23] with >99% of mitochondrial proteins encoded by nuclear genes that regulate mitochondrial biogenesis [24], organization [25], and structural integration with the flagellum [26].

Thus, mitochondrial architecture in sperm should not be viewed solely as a functional endpoint related to fertility, but rather as a downstream cellular manifestation of nuclear genetic regulation expressed during gamete formation. Because sperm within a single ejaculate can vary in mitochondrial properties such as sheath length [27], variation in mitochondrial abundance and organization provides a quantifiable, biologically grounded phenotype that reflects how nuclear alleles governing mitochondrial assembly are differentially realized among gametes within a sire.

Fluorescence Activated Cell Sorting (FACS) has become a powerful tool for high-throughput sorting of homogeneous cell populations into separate subpopulations based on physiological and morphological characteristics, as well as distinct fluorescent biomarkers. In cellular biology, flow cytometry can be used to evaluate cells based on characteristics such as size and shape [28], nuclear stain uptake [29], membrane integrity [30], and mitochondrial activity [31]. In livestock systems, flow cytometric sperm sorting has demonstrated that genetically distinct gamete populations can be isolated at scale, most notably through sex-sorted semen, establishing proof-of-principle that gamete-level partitioning of nuclear genetic material is both feasible and biologically meaningful [32–34].

However, sorting of sperm imposes mechanical and oxidative stress that can compromise sperm viability [35–38]. Variation in sperm resilience to sorting-induced stress [39] suggests that intrinsic cellular and genetic features influence how sperm tolerate perturbation, consistent with the presence of underlying nuclear genomic differences between sperm subpopulations. Such variation in sperm cellular phenotypes may stem from allelic biases established during spermatogenesis or selective pressures during sperm maturation.

During gametogenesis, alleles are transmitted to gametes in equal proportions according to Mendelian inheritance. However, deviations from this expected 1:1 ratio can occur. Allelic Ratio Distortion (ARD), or deviations from the expected 50:50 segregation of alleles in gametes, arise during meiosis [40] or selective pressures after ejaculation [41]. A previous study of ARD in boar sperm has analyzed unsorted, heterogeneous populations linking ARD to genes involved in spermatogenesis and fertilization, suggesting that post-meiotic selection may bias the sperm genome towards alleles that confer functional advantages [42].

Despite the growing recognition of functional heterogeneity among sperm, little is known about how specific mitochondrial phenotypes correspond to underlying genomic variation. During spermatogenesis and spermiogenesis, nuclear alleles orchestrate the assembly [43], length [44], and organization of the mitochondrial sheath [45], creating sperm subpopulations whose mitochondrial architecture reflects differential expression of nuclear genetic programs. Although paternal mitochondria themselves are not transmitted to offspring [46], the nuclear alleles that regulate mitochondrial assembly and organization are inherited [47–49], positioning sperm mitochondrial phenotypes as indirect but biologically meaningful markers of nuclear genome structure.

This study presents a novel integrative approach that combines Fluorescence Activated Cell Sorting of boar sperm based on mitochondrial abundance with whole-genome sequencing to investigate allelic ratio distortion (ARD) between distinct mitochondrial-defined subpopulations. Rather than framing mitochondrial phenotypes as determinants of fertility or fertilization, this work treats mitochondrial abundance as a potential phenomic readout of nuclear genomic architecture and within-sire allelic segregation. By resolving ejaculates into mitochondrial-high and mitochondrial-low sperm subpopulations using MitoTracker™ Green fluorescence, and interrogating nuclear allele frequencies within each fraction, we test the hypothesis that mitochondrial architecture serves as an indirect marker of genome-wide nuclear allelic structure.

This study represents one of the first efforts to directly link mitochondrial phenotyping with genome-wide nuclear allelic ratio distortion, establishing sperm mitochondrial architecture as a measurable, high-throughput indicator of within-sire nuclear genomic heterogeneity. This framework provides a potential foundation for precision breeding strategies that move beyond population-average genotypes by enabling direct characterization of allele transmission potential among gametes, supporting both gamete-level selection concepts and improved modeling of allelic segregation relevant to offspring genetic outcomes.

## Materials and Methods

### Semen collection and processing

Three commercial boars with known fertility from the same private boar stud were used in this study. The boars were housed one boar per pen, collected once per seven-day period, and fed a commercial boar diet to maintain weight. Semen doses in this study were in excess of industry production following established standard operating procedures and were not directly collected for this research. Boar semen was collected using the standard two-gloved hand technique. Only ejaculates with >80% motility were used. Sperm concentration was determined using a hemocytometer. The semen was immediately extended (e.g., < 5 min) a total of 5 times in a Preserve Xtreme extender, with the extender being within 2 °C of the semen, and delivered to Iowa State University the same day. Upon arrival, the temperature of the semen was annotated, and they were stored at 17 °C as previously described in [50]. The semen was then analyzed within 24 h of collection for concentration and motility using Computer-Aided Sperm Analysis (CASA) and adjusted for a final concentration of 30 million cells/1200 µL for each boar.

### Ethics statement

Ethical review and approval were not applicable for this study. This study involved the use of boar sperm samples, which were obtained from industry partners. The samples were not collected specifically for scientific research but were excess materials from standard production processes. The collection and handling of these samples adhered to standard industry protocols, designed to ensure ethical and humane treatment of the animals. Given the nature of the semen sample collection (a byproduct of routine production activities) and its alignment with standard agricultural practices, this study is exempt from Iowa State University Institutional Animal Care and Use Committee (IACUC) oversight.

### Computer-aided sperm analysis (CASA)

For concentration and motility, a 1000 µL aliquot of each sample was placed in a 1.5 mL Eppendorf tube and warmed for 20 min at 37 °C. Samples were then gently mixed thoroughly to ensure even distribution of cells, and then 3.5 µL of each aliquot was loaded onto a 20 µm disposable counting chamber from Minitube (Minitüb GmbH, Tiefenbach, Germany). Concentration and motility were then assessed using a Zeiss Axioscope 5 microscope (Carl Zeiss Microscopy, LLC, Oberkochen, Germany) fitted with a Basler ace ac2440−75uc camera (Basler AG, Ahrensburg, Germany) and a 10 × /0.25 A-Plan objective lens using Minitube AndroVision^®^ software (Reference Module: 12,500/1000, Tiefenbach, Germany). The condenser with aperture diaphragm was set at 1 and the 6-position filter wheel was set at 2. This CASA workflow was performed as previously described [50], with minor modifications for the present experimental design.

### Media

#### Porcine non-capacitation media (pNCM).

Non-capacitation media containing NaCl, KCl, NaH2PO_4_, Na lactate, MgCl_2_-6H_2_O, TL-HEPES, Na-pyruvate, sorbitol, gentamicin, penicillin G, polyvinyl alcohol (PVA), and glucose was prepared in 1000 mL of deionized water (ddH_2_O), as previously described [51]. Vacuum filtration was performed into an appropriate container, and the pH of the solution was adjusted to 7.20 ± 0.02 and stored at 4 °C until use.

### Reagents

#### Image-based flow cytometry and fluorescence activated cell sorting.

MitoTracker™ Green (M7514; Invitrogen, Waltham, MA, USA) was reconstituted in DMSO to a stock solution of 1 mM and further diluted 1:10 in DMSO as a sub-stock prior to staining. Hoechst 33342 (H33342; Calbiochem, 382065, San Diego, CA, USA) was reconstituted with ddH_2_O to a stock solution of 18 mM. Propidium iodide (AC440300010; Acros Organics, Geel, Belgium) was reconstituted with ddH_2_O to a stock solution of 1 mg mL^−1^.

### Flow cytometry probe staining and incubation

For FACS, sperm concentration was taken from CASA measurements to adjust concentration to 30 million cells/mL, centrifuged at 500 × g for 5 minutes, supernatant removed, and resuspended in 600 µL of pNCM with H33342, Propidium Iodide, and MitoTracker™ Green at a 1:1000 concentration using the above stock solutions. Samples were covered and incubated at room temperature for 30 minutes, then centrifuged at 500 × g for 5 minutes. The supernatant was removed, and sperm were resuspended in 1200 µL of room temperature pNCM in a 5mL polystyrene tube before sorting.

### Fluorescence activated cell sorting (FACS)

Cell sorting was performed using the BD FACSMelody™ Cell Sorter (BD Life Sciences, San Jose, CA, USA). The instrument was equipped with three spatially separated excitation lasers: 405 nm violet laser (40 mW), 488 nm blue laser (20 mW), and 640 nm red laser (40 mW). The fluidics system utilized a quartz cuvette flow cell with gel-coupled optics for optimal light collection and resolution. Sheath fluid consisted of 1x Gibco™ Phosphate-Buffered Solution (PBS) at pH 7.20. A 100 µm nozzle was used to ensure gentle handling of sperm cells. Flow speed was maintained at a setting of 10. Sample input was controlled via software-adjustable agitation at 200 rpm to keep cells suspended. Sorted populations were collected in 5.0 mL polystyrene tubes. The photomultiplier tube (PMT) settings for the H33342 gated population was set at 55. The sorting process was managed using BD FACSChorus™ Software (v1.4.3.0), which provided automated setup and optimization of droplet breakoff, stream alignment, and sort delay using BD FACS™ Accudrop beads. Instrument performance was verified daily using BD CS&T quality control beads to ensure consistent fluorescence sensitivity and alignment. The cell sorting protocol was adapted from a previously published method [39], with modifications described below.

Gating was performed sequentially, beginning with forward scatter (FSC) / side scatter (SSC) to identify sperm and exclude debris, followed by exclusion of doublets using FSC width (W) versus FSC height (H). From the single-cell population, Hoechst 33342-positive cells were selected, and within this population, propidium iodide-negative cells were gated to exclude dead or membrane-compromised cells. The final gating step resolved subpopulations based on MitoTracker™ Green fluorescence with cells sorted according to approximately the lowest one-third and highest one-third of the fluorescence intensity histogram, while excluding events associated with histogram tailing or irregular distribution artifacts (S1 Fig). Acquisition rates ranged between 5,000−7,000 events per second. The sort rate ranged from 800−1200 events per second. Samples sat no longer than 30 minutes in catch media while in the sorter for optimized throughput. After sorting, samples were taken off the sorter and placed in a swinging hinge centrifuge and spun down at 500 × g for 20 minutes, supernatant removed, and washed with 1x PBS a total of 2 times. All samples were handled under identical sorting and post-sorting conditions to minimize handling related variation. After the final washing, samples were centrifuged at 13,000 g × 5 minutes, supernatant removed, and frozen at −80°C until Genomic DNA extraction.

### Image-based flow cytometry (IBFC) data acquisition

A Cytek® Amnis® ImageStream®X MkII Image-Based Flow Cytometer (ISX, Fremont, CA, USA) fitted with a 40 × microscope objective and an imaging rate up to 2,000 events/s was used for analyzing and validating sorted sperm biomarker status. The sheath fluid was phosphate-buffered solution void of Mg²⁺ or Ca²⁺ with a pH of 7.20. The flow-core diameter and speed were 6 µm and 66 mm/s, respectively as previously described [39]. Raw image data were acquired using INSPIRE® software version 3.0. Per boar, 5 million sorted cells from both the low and high MitoTracker™ Green populations were resuspended in 100 µL of 1x PBS, and 10,000 events were acquired for imaging. In INSPIRE® data acquisition software, the following channels were collected: two bright-field channels (channel 1), MitoTracker™ Green (channel 2), propidium iodide (channel 5), side scatter (SSC; channel 6), and Hoechst 33342 (channel 7). The following lasers and power settings were used: 405 nm (to excite Hoechst 33342): 10 mW; 488 nm (to excite MitoTracker™ Green): 10mW; 561 nm (to excite propidium iodide): 100 mW; and 785 nm (SSC laser): 5 mW. SpeedBeads were used to ensure focus of the cells throughout acquisition.

### Image-based flow cytometry data analysis

Data were analyzed using IDEAS^®^ analysis software version 6.4. The gating approach used standard focus and single cell gating calculations created by IDEAS^®^ software. Image-based calculations were also used to discard spermatozoa laterally aligned with the camera, as opposed to anteriorly/posteriorly aligned, as previously described [52].

### Epifluorescence microscopy

Sperm subpopulations differing in mitochondrial abundance (low and high MitoTracker™ Green fluorescence) were sorted directly onto Fisherbrand™ Superfrost® precleaned microscope slides (25x75x1.0 mm; Fisher Scientific, Pittsburgh, PA, USA) using the BD FACS Melody Cell Sorter. Slides were covered with FisherFinest™ Premium cover glass (18x18 mm; Fisher Scientific, Pittsburgh, PA, USA) and were imaged immediately post-sort to prevent fluorophore fading.

Imaging was performed using a Leica DM6 Thunder Imager equipped with Computational Clearing Technology and controlled by Leica Application Suite X (LAS X; v.3.7.4.23463; Leica Microscope CMS GmbH, Wetzlar, Germany). The system was fitted with a K6 sCMOS camera (Leica Microscope CMS GmbH) and a 40x/0.65 objective lens. Fluorescence excitation and emission were optimized for MitoTracker™ Green (excitation 488 nm; emission 520 nm) and Hoechst 33342 (excitation 405 nm; emission 461 nm). All brightfield morphology images were acquired using differential interference contrast (DIC).

Image acquisition parameters (exposure time, gain, LED intensity) were standardized across samples. All images were collected using the Thunder Computational Clearing mode to enhance midpiece resolution and suppress background fluorescence. Prior to measurement, the segmented line measurement tool was calibrated using a stage micrometer to ensure accurate distance quantification. Mitochondrial sheath length was quantified in Leica LAS X (Thunder edition) using the segmented line measurement tool, and 500 cells were measured for each subpopulation between all three boars (≈160 individual cells per boar). Measurements were restricted to morphologically intact spermatozoa that were fully in focus and displayed a clearly defined head, midpiece, and tail, with a straight and continuous midpiece for accurate length quantification. Comparisons of mitochondrial sheath length between low and high mitochondrial fluorescence populations were conducted using a two-sided Welch’s t-test, with effect size calculated as Cohen’s d (non-pooled SD). Statistical analysis was performed in R (v4.4.1).

### Genomic DNA extraction

Approximately 30 million sperm cells per sample were collected by flow cytometric sorting, pelleted, and stored at −80 °C until extraction. For DNA isolation, frozen pellets were thawed on ice for 45 min, after which 500 µL of digestion buffer (10 mM Tris-HCl pH 8.0, 100 mM NaCl, 1 mM EDTA, proteinase K (10 mg/mL), and RNase A (10 mg/mL)) was added directly to the tubes. Samples were supplemented with 25 µL of 20% SDS and 25 µL of 1 M DTT. Samples were gently inverted a total of 10 times, then incubated overnight at 50 °C with shaking (180 rpm).

The following day, DNA was extracted with a 25:24:1 volume of phenol:chloroform:isoamyl alcohol and centrifuged at 13,000 × g for 15 min at 4°C. The aqueous phase was transferred to a new tube and DNA was precipitated with 900 µL of ice-cold isopropanol. At this stage, 1/10 total volume of 3 M sodium acetate was added, and samples were stored covered overnight at −20 °C. DNA was pelleted by centrifugation (13,000 × g, 5 min at 4°C), washed twice with 70% ethanol and once with 100% ethanol. Samples were then air-dried for a total of 2 hours and resuspended in 200 µL of RNase/DNase-free water. Samples were allowed to solubilize at room temperature for 24 hours prior to quantification.

### DNA quantification and dilution

DNA concentration and purity were measured using a NanoDrop Lite spectrophotometer (Thermo Scientific, Wilmington, DE, USA). Each sample was measured in tandem, and the values were averaged to obtain a final concentration. Based on the averaged values, DNA was diluted to a working concentration of 20–35 ng/µL in 30 µL of RNase/DNase-free water for submission to the Iowa State University DNA Facility for QUBIT quantification, library preparation, and sequencing.

### Whole-genome sequencing

DNA was quantified using a Qubit 4 Fluorometer (Invitrogen, Waltham, MA, USA) with the Qubit dsDNA BR Assay Kit (Q32853), following the manufacturer’s instructions. Genomic libraries were prepared using the NEBNext Ultra II FS DNA Library Prep Kit for Illumina (E7805L) according to the manufacturer’s protocol. Library quality was assessed using both Qubit and an Agilent 2100 Bioanalyzer with the High Sensitivity DNA Kit (5067−4626). Sequencing was performed at 30x coverage on an Illumina NovaSeq 6000 platform using an S1 flow cell, run in paired-end mode with 150 cycles (kit 20028317). Base calling and demultiplexing were performed using the Illumina bcl2fastq program. All whole-genome sequencing in this study was performed on bulk, population-level genomic DNA pooled from approximately 30 million sorted cells per fraction; no single-cell isolation or whole-genome amplification was used at any step, avoiding the coverage biases and allele dropout associated with low-input or amplified sequencing workflows.

### Bioinformatic processing and variant calling

Adapter trimming and quality filtering were performed with TrimGalore (v0.6.6), which implements Cutadapt [53] for adapter removal and quality reports were generated using FastQC (v0.11.9) [54] and MultiQC (v1.30) [55]. Filtered reads were aligned to the *Sus scrofa* 11.1 reference genome [56] using BWA-MEM (v0.7.17) [57]. SAMtools (v1.19.2) [58] was used to sort and index the aligned BAM files. Read groups were added and edited with Picard AddOrReplaceGroups, and duplicate reads were removed using Picard MarkDuplicates (v2.26.2; Broad Institute).

For each boar, high and low mitochondrial populations were merged while retaining individual population files. Statistics on read counts and coverage were assessed with SAMtools flagstat and depth (v1.19.2) [58]. Variant calling was performed using GATK HaplotypeCaller (v4.3.0) [59,60]. Hard filtering of variants was applied with GATK VariantFiltration (v4.3.0) [60] to remove low-confidence calls and reduce potential technical biases arising from sequencing and alignment artifacts. Variants were filtered using the following thresholds: quality by depth (QD < 2.0), Fisher strand bias (FS > 60.0), strand odds ratio (SOR > 3.0), mapping quality (MQ < 40.0), mapping quality rank sum (MQRankSum < –12.5), and read position rank sum (ReadPosRankSum < –8.0). Variants overlapping repetitive elements identified by a custom repeat-masked BED file were also removed. Only SNPs that passed all filter criteria were retained. Insertions and deletions were excluded from further analysis.

Final variant statistics were generated using bcftools stats (v1.19) [61]. Variants were compared between high and low mitochondrial populations relative to merged controls, and allele frequencies were assessed. After hard filtering, the merged population for each boar was further restricted to SNPs with allele frequencies between 0.4–0.6 to retain high-confidence heterozygous loci approximating the expected 1:1 allelic ratio in the diploid genome. This step established an internal baseline for detecting deviations in allele representation between mitochondrial fluorescence fractions. Overlapping SNPs between the merged population and each corresponding high and low mitochondrial population per boar were then identified using bcftools isec (v1.19) [61], and allele frequencies of the alternative allele were calculated independently for the high and low populations. Functional annotation of filtered variants was performed with SnpEff (v5.2) [62].

### Candidate SNP selection

For each boar, SNPs present in the merged population and both corresponding high and low mitochondrial subpopulations were retained for downstream analysis. The merged population was used as the heterozygote baseline, defined by a Reference (REF)/Alternative (ALT) allele frequency (AF) window of 0.40–0.60. All SNPs were required to have a minimum sequencing depth of 30 reads. For each Single Nucleotide Polymorphism (SNP), deviations in AF between high and low subpopulations were first evaluated using a 2-sided proportion Z-test on allele counts, analyzing all boars simultaneously. This step was used as a screening approach to prioritize candidate loci and therefore employed a comparison-wise significance threshold (p < 0.0001), and SNPs that did not pass this threshold were discarded. Allele counts were then evaluated independently within each boar using Fisher’s exact test. Multiple testing correction was then applied within each boar using the Benjamini–Hochberg method [63], and only SNPs with adjusted p ≤ 0.05 were retained. All analysis was performed in R software (v4.4.1).

### Gene ontology

Genes associated with candidate SNPs were subjected to ontology and pathway enrichment analysis using ClueGO (v2.5.10) [64] in Cytoscape (v3.10.3) [65]. All gene lists were analyzed together in a unified ClueGO run, with each boar-specific and shared gene set maintained as independent clusters for downstream analysis using *Sus scrofa* as the reference organism. The Biological Process (BP), Molecular Function (MF), and Cellular Component (CC) ontologies, as well as KEGG pathways, were tested for enrichment. Significance was assessed using a two-sided hypergeometric test with Benjamini–Hochberg correction, and terms with adjusted p-values ≤ 0.05 were considered enriched.

## Results

### Identification and validation of mitochondrial fluorescence-based sperm populations

**Fig 1:**
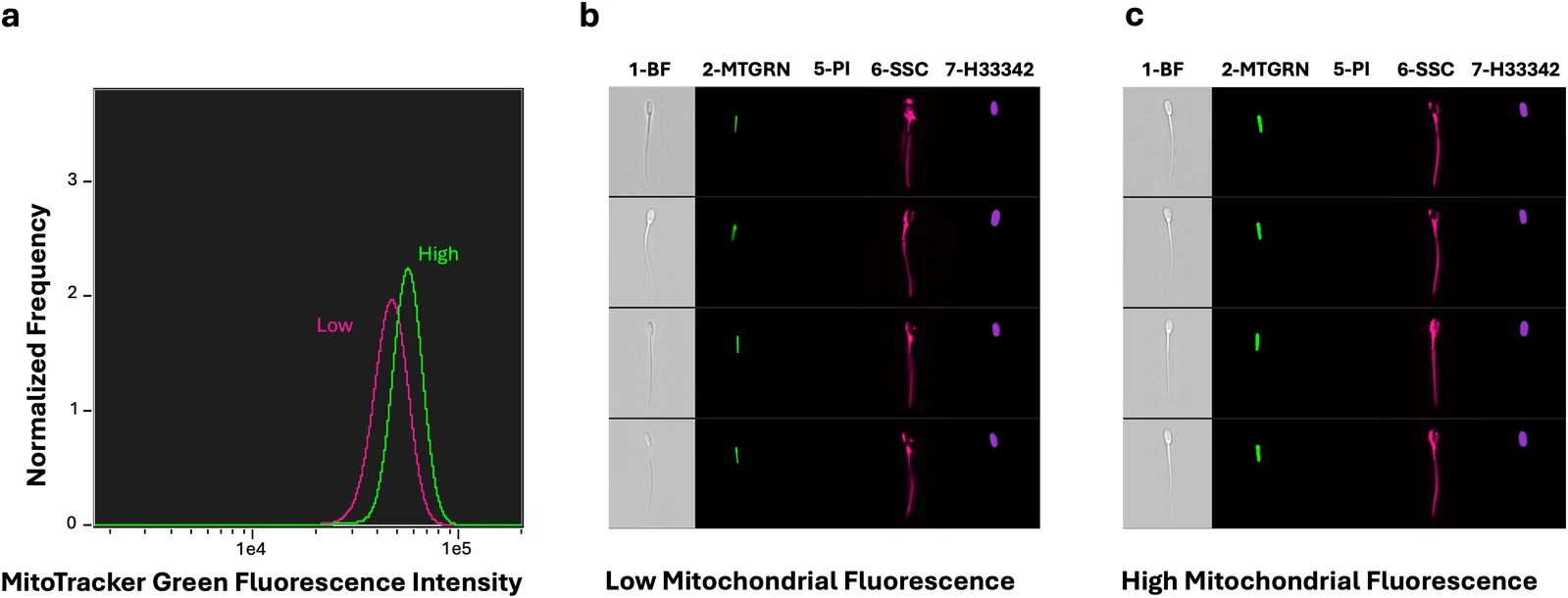
Flow Cytometric identification and fluorescence characterization of low and high mitochondrial sperm subpopulations. (a) Histogram overlays between the Low (magenta) and High (green) subpopulations, showing fluorescence of MitoTracker™ Green. (b-c) Representative images taken from the Cytek® Amnis® ImageStream®^X^ MkII image-based flow cytometer (Fremont, CA, USA) of low and high mitochondrial subpopulations after sorting. Columns are denoted by channel number and fluorescent probe, and rows are single-cell images of different spermatozoa. Channels used: (1) Brightfield (BF), (2) MitoTracker™ Green (MTGRN), (5) Propidium Iodide (PI), (6) Side-scatter (SSC), and (7) Hoechst 33342 (H33342). Examples of (b) low mitochondrial fluorescent spermatozoa and (c) high mitochondrial fluorescent spermatozoa that are H33342 positive and PI negative. Only one boar is displayed here for illustrative purposes.

In this study, image-based flow cytometry (IBFC) was utilized to assess the fluorescent intensity shifts between the two sorted high and low mitochondrial fluorescence subpopulations, with representative images from one boar shown in Fig 1. IBFC images show sperm from a single ejaculate captured across multiple channels. Brightfield imaging (Channel 1 – BF) provides a clear view of the sperm morphology, ensuring only structurally intact cells were analyzed. The MitoTracker™ Green probe (Channel 2 – MTGRN) localizes to the mitochondrial sheath, which is independent of membrane potential and can be used as a marker for mitochondrial mass [66], displays distinct fluorescence intensities between the low and high subpopulations, validating that sorting effectively separates cells based on mitochondrial content. Propidium Iodide (Channel 5 – PI), which serves as a marker for non-viable cells, revealed minimal fluorescence in both groups, confirming that the sorted subpopulations consist of viable, uncompromised spermatozoa. Hoechst 33342 (Channel 7 - H33342) was used to stain and visualize the sperm nucleus. Collectively, these multi-parametric analyses indicate mitochondrial fluorescence shifts between the two sorted subpopulations and support the robustness of the sorting strategy.

**Fig 2:**
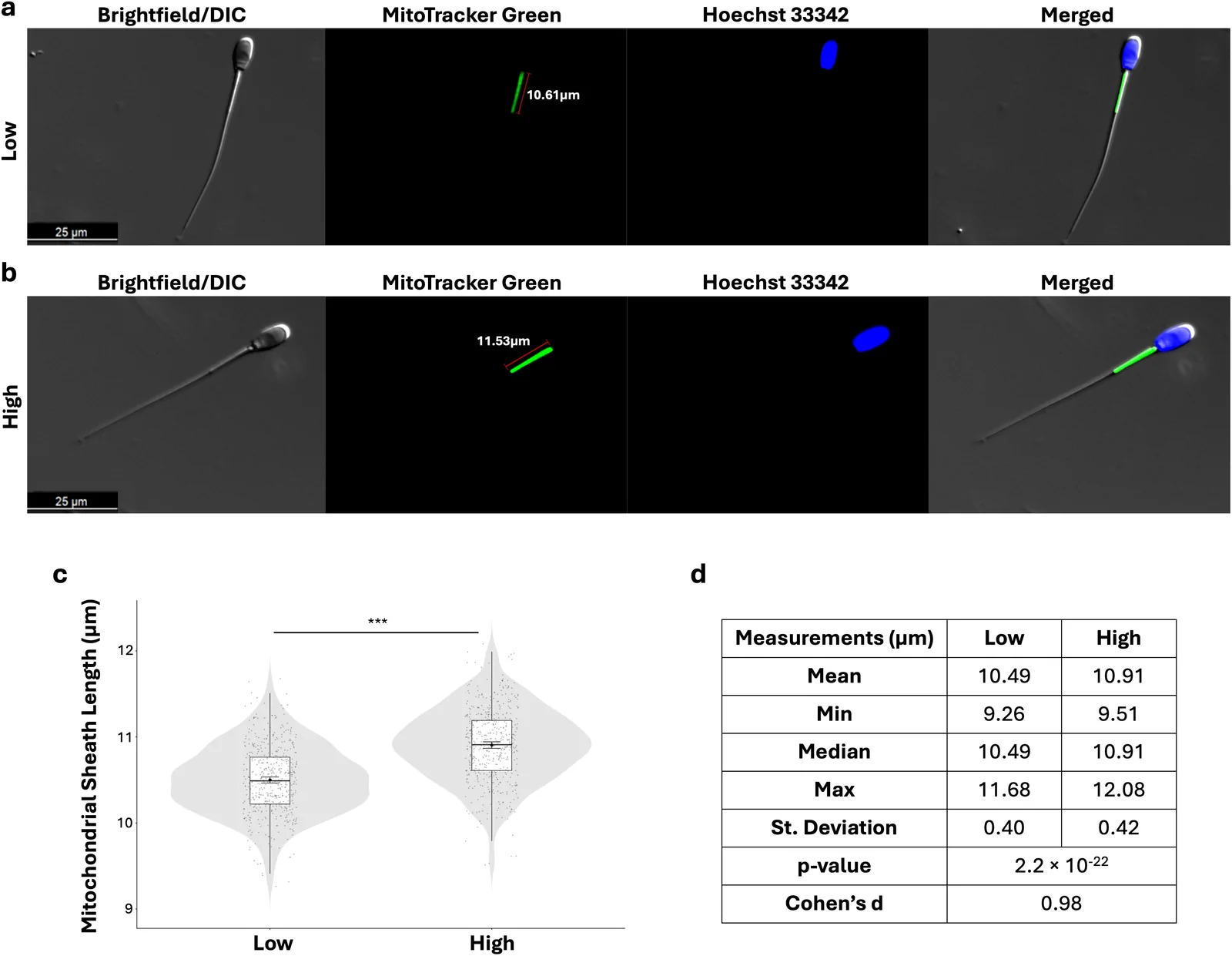
Morphological Validation of Low and High Mitochondrial Fluorescence-Defined Sperm Populations by Mitochondrial Sheath Length. (a-b) Representative spermatozoa from the (a) low and (b) high subpopulations following fluorescence-activated cell sorting. Columns show Brightfield/Differential Interference Contrast (DIC), MitoTracker™ Green fluorescence, Hoechst 33342 nuclear stain, and merged images. Mitochondrial sheath length was measured with representative measurements shown in µm and scale bar = 25 µm. (c) Violin plots with overlaid boxplots showing the distribution of mitochondrial sheath lengths for the low and high subpopulations. *** denotes p < 0.001 determined by Welch’s two-sided t-test. (d) Summary statistics for mitochondrial sheath length between the two subpopulations.

Spermatozoa sorted into low and high mitochondrial fluorescence populations, were further phenotyped to assess mitochondrial sheath length using quantitative epifluorescence microscopy (Fig 2). Representative post-sort images confirmed clear visualization of the mitochondrial sheath along the sperm midpiece. Mitochondrial sheath length was significantly greater in the high mitochondrial fluorescence population compared with the low population (Welch’s t-test: t = 15.48, df = 993.5, p = 2.2 × 10–22), with mean lengths of 10.91μm and 10.49μm, respectively. The mean difference of 0.42 μm was supported by a 95% confidence interval (0.35–0.46 μm) and a large effect size (Cohen’s d = 0.98, 95% CI: 0.85–1.11). Measurements were consistent across all three boars, indicating a reproducible structural distinction between the low and high mitochondrial fluorescence sperm populations. Fluorescence intensity data shown in S1 Table.

### Whole-genome sequencing of sorted mitochondrial fluorescence sperm fractions

Having confirmed that the sorted low and high mitochondrial populations represent biologically distinct and viable sperm subpopulations, we next performed whole-genome sequencing (WGS) on both sorted fractions from each boar. The low and high mitochondrial samples were pre-processed and aligned separately, and the resulting sorted and indexed BAM files were then computationally merged per boar. The merged dataset was filtered to retain only SNPs with allele frequencies between 0.4–0.6, providing a heterozygous proxy against which deviations in the two mitochondrial subpopulations could be compared and tested for heterogeneity of allelic frequencies between high and low populations, i.e., allelic ratio distortion (ARD) analysis.

**Fig 3:**
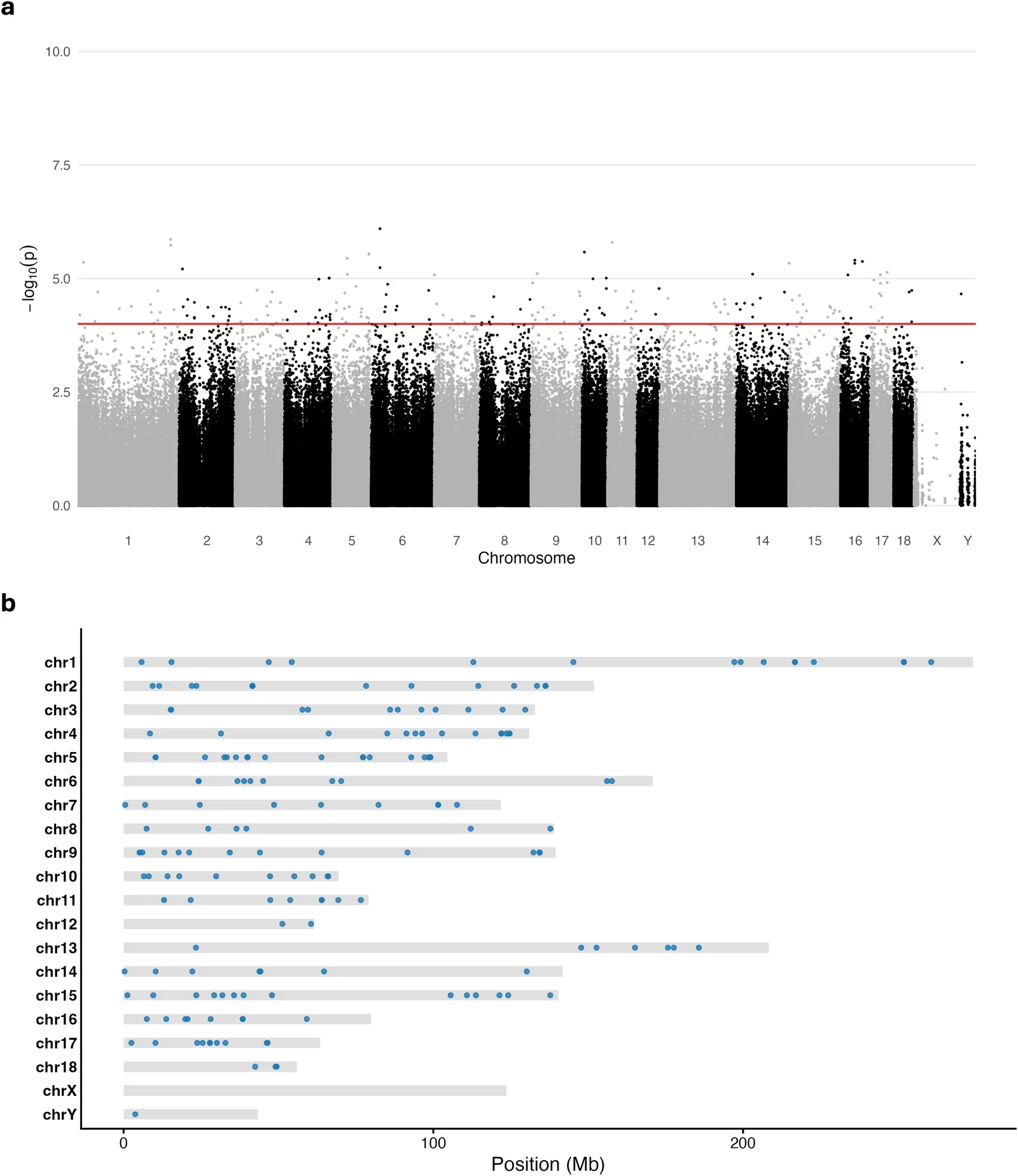
Screening of allelic ratio distortion across all three boars. (a) Manhattan plot showing the results of a two-sided proportional Z-test performed on the low and high mitochondrial subpopulations. The red horizontal line denotes threshold used for downstream candidate SNP selection (p < 0.0001, -log_10_(p) = 4). (b) Genome-wide mapping of Single Nucleotide Polymorphisms (SNPs) passing the initial comparison-wise threshold between mitochondrial fluorescence-defined sperm subpopulations. Each horizontal bar represents an individual chromosome (chr1-chr18, chrX, chrY), plotted by physical position (Mb). Blue points denote the positions of SNPs that passed statistical thresholds, while grey bars are scaled to represent physical length in the reference genome.

On average, 352.7 million paired end reads per sample were obtained (S2 Table), with up to 99.6% mapping to the *Sus scrofa* 11.1 reference genome. Duplicate reads across all three boars averaged 16.2% of total reads and were excluded from further analysis. Genome coverage ranged from 29–43× between the low and high mitochondrial subpopulations and from 65–75× for merged datasets across all three boars. On average, 7.4 million SNPs were detected across all three samples with 4.6 million overlapping heterozygous sites. After quality filtering, an average of 2.1 million SNPs across all subpopulations were retained for downstream analysis (S2 Table). After filtering, 182 SNPs passed the initial comparison-wise threshold (p < 0.0001) in the proportional Z-test (Fig 3), exceeding expectations under a null model of random allele segregation. These SNPs represent candidate loci with evidence of allele frequency divergence between high and low mitochondrial subpopulations.

A Fisher’s exact test using allele counts from the high and low mitochondrial subpopulations per boar was used to assess allelic distortion estimates. This ensured that SNPs identified in the proportional Z-test were not driven by a single boar but instead reflected true within-boar divergence between the mitochondrial phenotypes. Fisher’s exact tests were applied independently to each boar, with multiple testing correction performed using the Benjamini-Hochberg method [63] (S3 Table).

To evaluate how consistently ARD-associated loci appeared across individuals, we categorized all significant SNPs based on their presence in one, two, or all three boars (S4 Table). After filtering with both proportional Z-test, all 182 SNPs were confirmed with the Fisher’s exact test. Most candidate SNPs were boar-specific, with 35 SNPs detected in Boar 1, 27 SNPs detected in Boar 2, and 119 SNPs detected in Boar 3. Only one SNP was shared between Boar 1 and Boar 3, while no SNPs were shared between all three boars. These results indicate that allele-frequency divergence between mitochondrial fluorescence-defined sperm fractions was detected primarily within individual boars.

**Fig 4:**
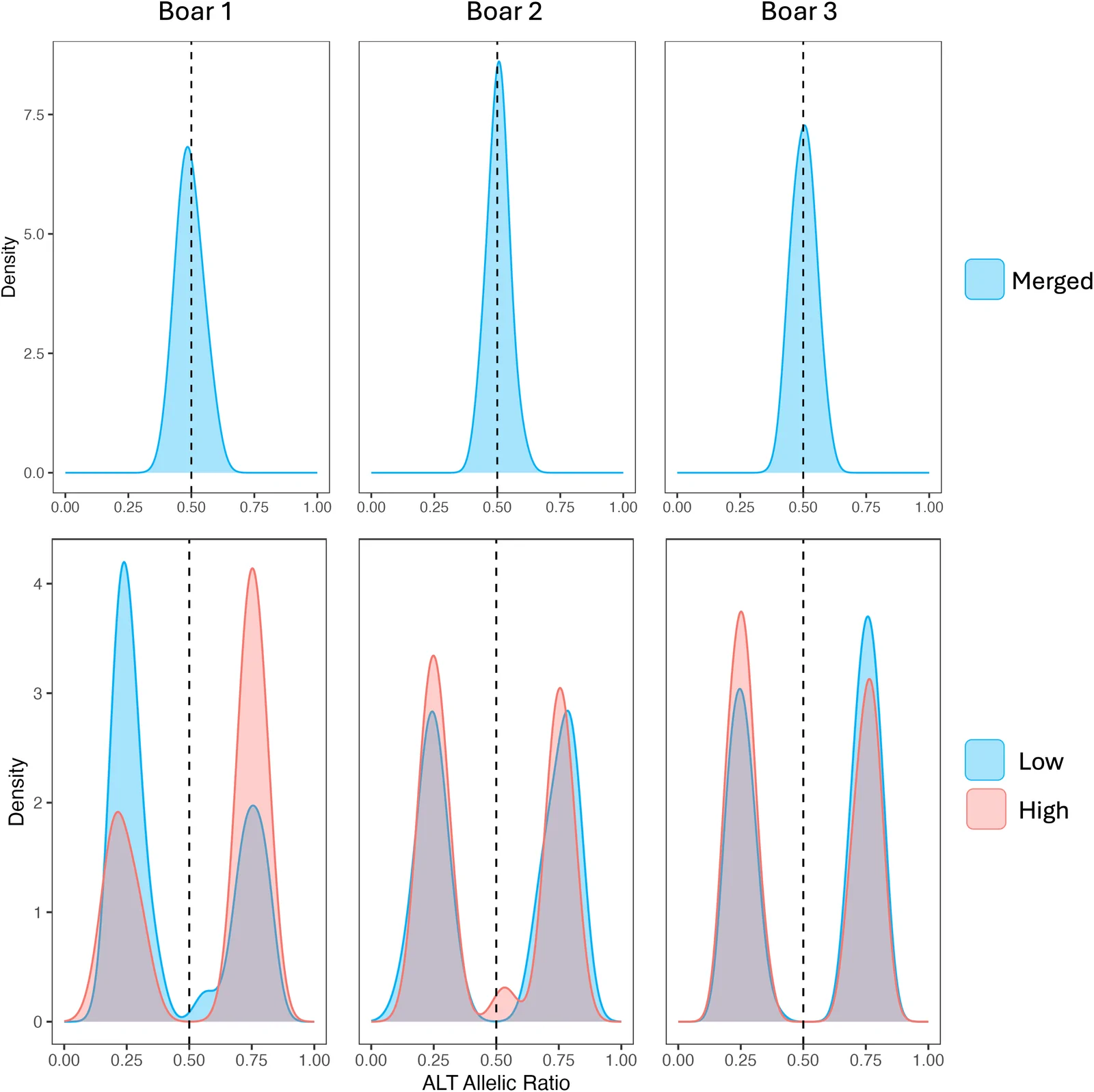
Allele frequency distributions of significant ARD-associated SNPs. (a-b) Density plots of allele frequency distributions between subpopulations and merged populations per boar from the 182 significant SNPs found in ARD. (a) Allelic ratio distributions for the heterozygous merged dataset. (b) Distributions of the alternative (ALT) allele ratios for the low and high mitochondrial subpopulations per boar. Low mitochondria population is shown in blue, high mitochondria population is shown in red.

To visualize how allelic ratios differed between mitochondrial subpopulations, we examined the allele frequency distributions for each boar for the 182 SNPs that passed the two-sided proportional Z-test between all boars and Fisher’s exact test within each boar (Fig 4). The low and high mitochondrial subpopulations showed pronounced and opposing shifts in allele frequency at ARD-associated loci mostly outside of 0.4–0.6. In contrast, the merged populations displayed a narrow distribution centered between 0.4–0.6 for all three boars, confirming that only heterozygous SNPs were carried forward in the merged population.

### Gene ontology enrichment

To identify biological processes represented among genes annotated to ARD-associated candidate variants, pathway and Gene Ontology enrichment analysis was performed using the combined candidate gene list (S5 Table). Among several enriched terms with a q < 0.05, candidate loci were associated with regulation of reproductive processes, supported by variants in *CCNB2, RLN2, SFRP1, SULF1,* and *ZFPM2*. Additional shared enrichment was observed in pathways related to cellular response to growth factor stimulus, supported by variants in *ADAMTS12, RASL11B, ROBO1, SFRP1,* and *USH2A*. Additional pathways related to embryo development were supported by variants in *APBA1, CCNB2, IFT52, MFNG, PLPP4, PLXNA2, RESP18, RLN2, SFRP1, SOX11, SULF1,* and *ZFPM2*. Because allelic distortion signals were largely boar-specific at the variant level, this pathway enrichment analysis reflects convergence at the level of biological processes rather than shared individual SNPs. The enriched pathways were supported by different variants of genes in different boars that converged on common functional themes. Accordingly, the Gene Ontology results should be interpreted as hypothesis-generating functional annotation of genes associated with allele-frequency divergence between mitochondrial fluorescence-defined sperm fractions.

## Discussion

Sorting sperm based on MitoTracker Green fluorescence intensity resolved two sperm subpopulations with differing mitochondrial abundance, reflected by relative fluorescence intensity and midpiece length, representing distinct mitochondrial phenotypes. These mitochondrial phenotypes were independently validated by image-based flow cytometry and epifluorescence microscopy, confirming consistent differences in mitochondrial distribution and midpiece architecture between subpopulations.

Although MitoTracker™ Green fluorescence is commonly used as a membrane potential-independent probe, fluorescence intensity may still be influenced by dye uptake, staining efficiency, mitochondrial membrane environment, or other cell-level variation. To minimize the influence of these potential artifacts, subpopulations were sorted using predefined gates that exclude the far left and right tails of the main fluorescence distribution, as shown in S1 Fig. Additionally, all samples were stained, sorted with comparable throughput timing, and processed under identical conditions to minimize dye and handling related technical variation. Sorted phenotypes were further evaluated using image-based flow cytometry and epifluorescence microscopy for downstream analysis.

The defined subpopulations were associated with differences in boar-specific nuclear genomic composition, as whole-genome sequencing revealed significant allelic ratio distortion at a subset of heterozygous loci. While the initial proportional Z-test was applied as a comparison-wise threshold across a large number of loci, the number exceeding this threshold was greater than expected under a null model of random allele segregation. Consistent with this, the observed allele frequency shifts indicate non-random segregation of nuclear alleles between mitochondrial-defined sperm fractions. Gene ontology enrichment of the combined candidate gene set identified biological processes related to cellular organization, extracellular matrix remodeling, cell motility, growth factor response, reproductive regulation, and developmental signaling (q < 0.05). These patterns suggest that genes annotated to candidate loci are involved in broad cellular and developmental processes relevant to sperm formation and structure. Overall, these findings suggest that mitochondrial fluorescence-defined sperm fractions are associated with differences in nuclear allele representation within ejaculates.

When mitochondrial-high and mitochondrial-low subpopulations were analyzed together as a homogeneous mixture, allele frequencies remained centered near the expected 0.5 ratio, consistent with Mendelian expectations for pooled gametes. In contrast, phenotypic partitioning by FACS on mitochondrial fluorescence intensity revealed pronounced and opposing allele frequency shifts between subpopulations, indicating that allelic distortion is masked when ejaculates are treated as unstratified populations. These findings suggest that within-sire nuclear genomic structure can be partitioned across distinct sperm phenotypes, and that resolving this structure requires integration of cellular phenotyping with analysis across the genome.

Although allelic ratio distortion signals were detected primarily within individual boars, one candidate SNP was shared between two boars (Boar 1 and Boar 3). No candidate SNPs were detected across all three boars after applying a minimum 30-read depth threshold. The question addressed by this analysis is whether sperm subpopulations defined by mitochondrial abundance carry nuclear alleles in the same proportions or in different ones, and it is resolved within an ejaculate rather than across animals. Allelic ratio distortion can be evaluated only at loci that are heterozygous within a given sire, so each boar contributes its own set of testable loci, and because the mitochondrial sheath is assembled during spermiogenesis within each male, the informative contrast is between subpopulations of the same ejaculate. Evaluated in this way, candidate loci were recovered in all three boars (35, 27, and 119 in Boars 1, 2, and 3, respectively), while the identity of those loci varied between animals as expected for unrelated sires. The limited cross-animal recurrence therefore follows from the structure of the analysis rather than from instability of the result, although it may additionally reflect individual-specific genomic architecture, the limited number of animals, and the conservative nature of the candidate SNP filtering strategy. As a preliminary analysis, candidate SNPs were also evaluated at a less stringent minimum depth threshold of 20 reads, which identified 379 candidate SNPs (S2 Fig.), including 64 SNPs shared between 2 or more boars and one SNP shared across all three boars (S6 Table). However, because this study represents an early proof-of-concept with a limited number of boars, the lower read depth increases uncertainty in allele-frequency estimation, therefore the 30-read threshold was used for primary interpretation. Additional boars would increase the ability to determine whether candidate loci detected here are truly-boar specific or represent recurrent allele-frequency differences.

The candidate SNP shared between Boar 1 and Boar 3 was annotated to CDH18, a member of the classical cadherin family with emerging relevance to male germ-cell biology. CDH18 has been reported in neonatal porcine prospermatogonia and murine spermatogonial stem cells, suggesting this adhesion-associated gene may participate in early germ-cell organization and maintenance [67]. More broadly, cadherins mediate calcium-dependent cell-cell adhesion [68] and contribute to the organization of testicular and gamete-associated cellular interactions [69–71]. While CDH18 represented the only candidate locus detected in more than one boar at the 30-read threshold, the remaining candidate SNPs were primarily boar-specific, allowing within-boar patterns of nuclear allele representation to be evaluated across genes with relevance to sperm structure, cellular signaling, mitochondrial regulation, and breeding-associated biological traits.

Several Boar-1 specific candidate loci were annotated to genes with biological relevance to sperm structure, cellular signaling, and developmental processes important to breeding outcomes. IFT52 encodes an intraflagellar transport protein involved in ciliary and flagellar assembly [72], making it a particularly relevant candidate in the context of sperm tail organization and motility-associated structure. STIM1 encodes a calcium-sensing protein involved in calcium entry, a signaling process relevant to fertilization-associated calcium dynamics [73,74]. Finally, LIN9 is involved in cell cycle regulation and transcriptional control of genes required for mitotic [75] and meiotic progression [76], linking this candidate locus to developmental competence and early embryogenesis [77]. These Boar 1 candidate genes provide a focused example of allele-frequency differences involving genes related to sperm structure, signaling and developmental regulation.

Within Boar 2, several candidate loci were annotated to genes involved in germ cell differentiation, endocrine signaling, and protein-interaction. SPERT (also known as CBY2 and Nurit) is a spermatid-associated gene expressed during late spermatogenesis [78,79], placing this candidate locus within a nuclear gene involved in male germ-cell development. ROBO1 encodes a receptor in the Slit/ROBO signaling pathway, which has been shown to regulate Leydig cell steroidogenic signaling and intratesticular testosterone concentrations [80]. Polymorphisms in this gene are associated with reduced damage from cryopreservation in pigs [81]. TTC4 encodes a tetratricopeptide repeat-containing protein associated with protein folding and DNA replication [82], and previous studies show TTC4 plays a role in steroid receptors expressed in the testis [83] and is associated testicular hypoplasia in cattle [84]. The Boar 2 candidate genes add a distinct set of germ cell, endocrine, and protein-regulatory processes to the broader pattern of within-boar allele-frequency divergence.

Within Boar 3, candidate loci were annotated to genes with established roles in mitochondrial quality control, sperm flagellar structure, ciliary motility, and lipid metabolism. PRKN encodes Parkin, an E3 ubiquitin ligase involved in PINK1/PARKIN-mediated mitophagy [85] and mitochondrial quality control pathways [86], linking this candidate locus directly to nuclear regulation of mitochondrial maintenance. SPAG17 encodes sperm-associated antigen 17, a flagellar structural protein required for normal spermatogenesis and sperm flagellar organization [87], and is associated with the regulation of reproductive aging in mice [88]. LRRC6 (also known as DNAAF11) encodes a leucine-rich repeat-containing protein involved in motile cilia function and dynein arm assembly, with disruption of LRRC associated with impaired sperm motility and flagellar defects in humans [89,90]. ELOVL6 encodes an elongase involved in long-chain fatty acid metabolism and has been associated with lipid composition [91] and meat-quality traits in livestock species [92,93]. These Boar 3 candidate genes highlight nuclear loci connected to mitochondrial quality control, sperm structural organization, motile cilia function, and lipid-related production traits within these mitochondrial fluorescence-defined sperm fractions.

Previous work has provided important foundations for understanding how allelic ratio distortion can be detected in boar sperm and linked to genes involved in spermatogenic processes when ejaculates are analyzed as aggregate populations [42]. Critically, treating ejaculates as homogeneous populations limits resolution of how allelic distortion may be distributed across phenotypically distinct sperm subpopulations derived from the same nuclear genome. In parallel, a broader body of literature has examined transmission ratio distortion (TRD) using offspring-based genotypes across multiple mammalian species [94–97]. Such approaches inherently integrate additional biological processes beyond gametogenesis, including fertilization dynamics [98], maternal genetic effects [99], and early embryonic development [100]. As a result, TRD-based inference complicates isolation of sperm-intrinsic sources of allelic bias, highlighting the value of approaches that directly interrogate gamete-level genomic structure without these confounding effects.

Although individual sperm within an ejaculate are expected to differ genetically as a consequence of meiosis and recombination, whether specific nuclear alleles are systematically enriched within distinct sperm phenotypes remains poorly understood. The observation that nuclear alleles partition non-randomly across mitochondrial-defined subpopulations raises the possibility that cellular phenotype may act as a potential functional stratifier of allele transmission potential. Although the present study does not assess fertilization outcomes, this framework invites future investigation into whether specific mitochondrial phenotypes exhibit differential tolerance to technological interventions such as cryopreservation or sorting, or whether particular allelic configurations are preferentially represented among gametes that successfully fertilize and support embryonic development. While the conservative 30-read threshold emphasized adhesion and development-associated loci, several nearest-gene annotations among these candidate loci also included metabolic and muscle-associated genes, including members of the insulin/IGF signaling axis such as IRS2 and IGFBP3 [101,102].

Given the limited number of animals and moderate sequencing depth, these results establish a proof-of-concept framework for resolving allele representation across mitochondrial-defined sperm subpopulations within a sire. This framework provides a basis for future studies that pair mitochondrial-defined genomic differences with fertilization outcomes, CASA-derived motility traits, larger sire populations, and deeper sequencing to better define their functional significance. Integrating subcellular phenotyping with genomic analysis may therefore provide a mechanistic bridge between gamete-level heterogeneity and transmission dynamics in both evolutionary and applied breeding contexts. Beyond the specific loci reported here, the finding itself is the contribution: within an ejaculate, sperm that differ in mitochondrial abundance differ in nuclear allele representation. Because the mitochondrial sheath is assembled during spermiogenesis under nuclear genomic control, this subcellular phenotype appears to retain information about the nuclear genome of the spermatid in which it formed. To our knowledge, such a relationship has not previously been described in any species. As the comparison is internal to a single sire, it also raises the prospect of within-sire gamete selection, in which an insemination dose is enriched for a phenotypically defined fraction of one male’s ejaculate. Ultimately, this framework could enable within-sire gamete selection for economically relevant offspring traits, including terminal production traits, by enriching sperm subpopulations associated with favorable nuclear alleles.

## Conclusion

Sperm within an ejaculate are expected to differ genetically as a consequence of meiosis and recombination, but these differences have not generally been considered resolvable at the level of individual cell phenotype. The present results indicate that they are not randomly distributed with respect to cellular phenotype: in the three boars examined, subpopulations differing in mitochondrial abundance differed in nuclear allele representation. The mechanism linking mitochondrial architecture to allelic segregation remains to be determined. By resolving ejaculates into mitochondrial-high and mitochondrial-low fractions and interrogating their nuclear genomes, we show that variation in mitochondrial architecture reflects underlying allelic segregation established during spermatogenesis and spermiogenesis. The observation of allelic bias across functionally related gene networks supports the concept that sperm phenotypic heterogeneity is rooted in structured genomic differences rather than stochastic variation. These findings aim to establish sperm mitochondrial architecture as a scalable phenomic readout of within-sire nuclear genomic heterogeneity and provide a potential framework for integrating gamete-level phenotypes with allelic ratio distortion analyses. More broadly, this approach enables empirical characterization of allele transmission potential among gametes, advancing precision breeding strategies that move beyond population-average genetic expectations.

## Supporting information

S1 Table Sequencing Metrics and Variant Summary of Mitochondrial Fluorescent-Sorted Sperm Subpopulations Across Three Boars. Whole-genome sequencing summary statistics for Low, High, and Merged mitochondrial fluorescence subpopulations across three boars.
(XLSX)

S2 Table Epifluorescence Microscopy Intensity Data. Descriptive statistics summarizing image-based fluorescence metrics for Low and High mitochondrial fluorescence sperm subpopulations.
(XLSX)

S3 Table Shared High-Confidence SNPs Exhibiting Allelic Frequency Shifts Between Mitochondrial Subpopulations. List of filtered heterozygous SNPs identified in merged populations and exhibiting allele frequency differences between Low and High mitochondrial fluorescence sperm fractions.
(XLSX)

S4 Table Distributions of Significant SNPs Across Individual and Combined Boar Comparisons. Summary of the number (n) of significant SNPs detected within individual boars and shared combination of boars.
(XLSX)

S5 Table Gene Ontology Enrichment of Shared ARD-Associated Genes Across Mitochondrial Subpopulations. Gene Ontology enrichment results for genes associated with significant allele frequency shifts between low and high mitochondrial fluorescence sperm fractions. Terms are annotated by composite list between all three boars and contributing genes are listed. Reported terms include biological processes and molecular functions, corresponding with raw p-values and Benjamini-Hochberg-corrected p-values.
(XLSX)

S6 Table Shared Genes with minimum depth of 20 reads per allele from preliminary analysis. List of filtered heterozygous SNPs identified in merged populations and exhibiting allele frequency differences between Low and High mitochondrial fluorescence sperm fractions with a 20 reads per allele filter.
(XLSX)

S1 Fig Gating Strategy for Isolation of Low and High Mitochondrial Fluorescence Sperm Subpopulations by Fluorescence-Activated Cell Sorting. (a) Initial gating of all recorded events based on forward scatter area (FSC-A) versus side scatter area (SSC-A) to define the general sperm population and exclude debris. (b) Side scatter height (SSC-H) versus side scatter width (SSC-W) was used to isolate singlet events and remove doublets and aggregates (SSC Singlets). (c) Further refinement of singlet sperm was performed using forward scatter height (FSC-H) versus forward scatter width (FSC-W) to ensure single cell identity (FSC Singlets). (d) H33342 positive cells were selected based on Hoechst 33342 fluorescence (H33342+), excluding unstained or non-nucleated events. (e) Cells that were MitoTracker Green positive (MTGRN+) and propidium iodide negative (PI-) were selected in a density plot to ensure only viable MitoTracker Green stained cells were sorted. (f) Final separation of mitochondrial subpopulations was achieved by histogram gating of MitoTracker Green fluorescence intensity within the MTGRN^+^/PI^-^ population.
(TIFF)

S2 Fig Preliminary Screening of allelic ratio distortion across all three boars at 20 read minimum threshold. (a) Manhattan plot showing the results of a two-sided proportional Z-test performed on the low and high mitochondrial subpopulations. The red horizontal line denotes threshold used for downstream candidate SNP selection (p < 0.0001, -log_10_(p) = 4). (b) Genome-wide mapping of Single Nucleotide Polymorphisms (SNPs) passing the initial comparison-wise threshold between mitochondrial fluorescence-defined sperm subpopulations. Each horizontal bar represents an individual chromosome (chr1-chr18, chrX, chrY), plotted by physical position (Mb). Blue points denote the positions of SNPs that passed statistical thresholds, while grey bars are scaled to represent physical length in the reference genome.
(TIFF)
